# Beyond Pathogenesis: The Nematode Immune Network as the Arbiter of a Host–Virus Truce

**DOI:** 10.3390/v17111485

**Published:** 2025-11-08

**Authors:** Emma Xi, Tan Meng, Hanqiao Chen

**Affiliations:** 1 Department of Biosciences, Rice University, 6500 Main Street, Houston, TX 77030, USA; ex5@rice.edu; 2 Department of Animal Sciences, University of Illinois Urbana-Champaign, 1201 W Gregory Dr, Urbana, IL 61801, USA; tanmeng2@illinois.edu

**Keywords:** nematode, *Caenorhabditis elegans*, Orsay virus, antiviral immunity, RNA interference (RNAi), intracellular pathogen response (IPR)

## Abstract

The phylum Nematoda is host to a vast and diverse virosphere, yet severe viral diseases are rarely observed. This paradox between pervasive infection and limited pathology suggests the existence of a highly effective host–virus “truce”. In this review, we argue that this truce is not a result of viral attenuation but is actively arbitrated by a multi-tiered host immune network, whose primary characteristic is not destructive power but exquisite cost–benefit management. We deconstruct this network into two functional tiers. The first, the “effector layer”, comprises a diverse arsenal of antiviral pathways, including RNA interference (RNAi), the Intracellular Pathogen Response (IPR), and other direct-acting mechanisms. The second, the “regulatory layer”, acts as a command hub, integrating internal physiological states—such as metabolism and aging—with external threat signals to orchestrate a proportional defense, thereby mitigating the high fitness costs of immunity. Understanding this intricate network is critical, as it not only explains the dynamics of infection within nematodes but also has profound implications for a broader medical landscape, particularly through the “Trojan Horse” effect, where nematode-borne viruses might elicit immune responses in their final vertebrate hosts. Together, these insights provide a unified framework for studying nematode–virus interactions and for comparing antiviral strategies across metazoans.

## 1. Introduction

For a long time, viruses were considered extremely rare in the vast phylum of Nematoda. This perception was largely rooted in the fact that the primary model organism, *Caenorhabditis elegans*, is maintained under standard laboratory conditions in a ‘virus-free’ or ‘naïve’ state; indeed, this lack of a known pathogen was a significant barrier to antiviral research. This paradigm was profoundly changed by the landmark discovery of Orsay virus, the first natural viral pathogen of *C. elegans*, which was identified not in a lab but in a *wild* isolate in 2011 [1]. Crucially, this foundational study also demonstrated that infected lines could be ‘cured’ via bleaching, establishing a clean, virus-free baseline that allows for the study of de novo infection and the *induction* of host immune pathways [1]. This finding catalyzed a new era of research, and subsequent advances in high-throughput sequencing have shattered the old paradigm, revealing a previously unknown, vast and diverse nematode virosphere. For instance, a meta-transcriptomic study of soil nematode communities discovered over 150 RNA viruses, the vast majority of which were new species, and a systematic survey of 41 parasitic nematodes identified 91 RNA viruses belonging to 24 families in approximately 70% of the species analyzed [2,3]. Despite this “omnipresent virus” backdrop, severe, devastating diseases in nematodes are rarely reported [4,5]. This striking contrast between pervasive infection and limited pathology constitutes a central paradox in modern nematode virology and impels an investigation into the underlying mechanisms.

This host–virus “delicate truce” appears to manifest in two principal patterns. The first is “controlled pathogenicity,” exemplified by the *Caenorhabditis*-virus model systems. In these cases, viral infection causes clear and dramatic intestinal symptoms, such as cytoplasmic disorganization and nuclear degeneration, a pattern common to this clade of viruses [1,6,7]. Structurally, Orsay virus is a non-enveloped virus with a T = 3 icosahedral capsid composed of 180 copies of its capsid protein, which form 60 trimeric spikes on the viral surface [8]. However, despite these severe intestinal symptoms, at the organismal level, this cellular damage translates to only a subtle fitness cost, such as slowed progeny production, rather than a significant impact on lifespan or total brood size [1]. Seminal studies have demonstrated the crucial role of the host immune system in this mitigation, as the infection phenotype becomes significantly more severe in its absence. Particularly, the RNA interference (RNAi) pathway is a major contributor to containing the infection [1]. The second, more common pattern is “persistent tolerance,” observed predominantly in parasitic nematodes. Pioneering metatranscriptomic studies revealed that species like *Heterodera glycines* and *Globodera pallida* harbor numerous, highly abundant RNA virus genomes [4,5,9]. These viral sequences are widespread and actively replicating yet uncorrelated with host virulence, leading to the compelling hypothesis of a stable co-evolutionary equilibrium maintained by vertical transmission [4,5,10]. This initial evidence, however, was primarily genomic. More direct support for active viruses has emerged from work showing that viruses within filarial nematodes, such as *Brugia malayi* and *Onchocerca volvulus*, elicit specific antibody responses in their vertebrate hosts, confirming the presence of expressed viral proteins [2]. Therefore, despite progress, the precise modes of viral transmission in most parasitic nematodes remain largely uncertain.

How, then, is this truce maintained across such diverse host–virus interactions? Is it due to viral restraint of host immunity? While viral restraint may play a role, growing evidence points to host immunity as the primary arbiter. The immune response is not merely defined by its destructive power. Recent dynamic transcriptomic studies of an ongoing infection have revealed that activating the immune response is metabolically “expensive,” causing massive shifts in host gene expression and resource allocation [11]. Therefore, a key characteristic of an effective immune system is its ability to manage these internal costs. To explore this principle, this review will deconstruct the architecture of nematode antiviral defense into two functional tiers: the “effector layer,” comprising the frontline combat units, and the “regulatory layer,” the command center responsible for orchestrating the defense. Given that our molecular understanding is most advanced in the model organism *C. elegans*, we will primarily use it as a framework to build our understanding while drawing critical comparisons to parasitic nematodes wherever possible. By dissecting this network, we aim to reveal how nematodes achieve a delicate balance between effectively clearing viruses and maintaining their own physiological homeostasis. This understanding is critical as the complexity of these interactions extends beyond a simple bilateral relationship. The discovery of viruses within nematodes that parasitize vertebrates, and the subsequent finding that these nematode viruses are immunogenic in their final vertebrate hosts, including humans, highlights that the outcome of the nematode’s internal immune struggle has profound implications for a far broader ecological and medical landscape [2,12].

## 2. The Arbiter of the Truce: Architecture of the Nematode Antiviral Defense Network

The preceding introduction established a central paradox of nematode virology: a world teeming with viruses, yet where overt and severe disease is the exception rather than the rule [2,3,4,5]. This observation raises a critical question: How do nematodes maintain this “delicate truce” in the face of such pervasive viral pressure? As we have seen, this truce manifests in at least two distinct patterns: the “controlled pathogenicity” observed in *C. elegans*, driven by a powerful immune response, and the more enigmatic “persistent tolerance” widespread in parasitic nematodes.

The molecular basis for these divergent outcomes is a central question in the field. While “controlled pathogenicity” appears to be the result of a potent immune arsenal clashing with the virus, the profound stability of “persistent tolerance” is less clear. It could stem from a fundamentally different host immune strategy that prioritizes tolerance over clearance to avoid self-damage, a co-evolutionary path where viruses have attenuated their virulence to ensure stable vertical transmission, or a delicate combination of both.

The answer to how these different strategies are orchestrated lies not in viral restraint, but within a multi-layered host immune network, whose architecture is finely tuned for management and control. To understand this architecture, this chapter will deconstruct the nematode’s defense system into its two fundamental tiers. The first is the “Effector Layer”—a diverse arsenal of frontline combat units that directly engage and suppress viral threats. The second is the “Regulatory Layer”—a command and control center that integrates physiological cues with threat signals to orchestrate a proportional and cost-effective defense. By examining these two interconnected tiers, we can begin to appreciate the molecular wisdom that allows nematodes to achieve different forms of truce while preserving their own fitness.

### 2.1. The First Tier: The Direct-Acting Effector Arsenal

The frontline of the nematode’s defense is a diverse “armory” of effector pathways, each with distinct mechanisms and activation kinetics, poised to counter the varied threats posed by different viruses [1,2]. This arsenal exerts direct pressure at different stages of the viral life cycle to contain infections and maintain the states of “controlled pathogenicity” or “persistent tolerance” observed in nature. First, structural proteins of the cytoskeleton and extracellular matrix have been found to form physical barriers to infection [13]. Upon viral entry, the host’s defense pivots to inhibiting replication and gene expression. The most well-characterized of these is RNA interference (RNAi), a precision-guided system providing robust protection [1]. This is complemented by other programs like the broad transcriptional shield of the Intracellular Pathogen Response (IPR), *C. elegans*-specific adaptation evolved to combat intracellular threats, and the specialized CDE-1-mediated uridylation pathway [14,15]. Intriguingly, this effector arsenal is further augmented by the co-opting of proteins from seemingly unrelated cellular processes. For instance, core components of the Programmed Cell Death (PCD) machinery have been shown to possess non-canonical antiviral functions [16]. Finally, classic antimicrobial effectors (AMEs) are also recruited to the antiviral battlefield to restrict viral spread [11]. This powerful and diverse arsenal, however, does not operate without consequence. As recent studies have shown, its activation incurs significant metabolic and cellular costs. This highlights a critical trade-off, distinguishing the external ‘cost of infection’ (the damage *from* the virus) from the internal ‘cost of immunity’ (the energetic price paid *by* the host to fight it). This necessity to manage its own expensive defense necessitates a higher level of coordination, which is orchestrated by the regulatory layer [11].

#### 2.1.1. Barricading the Gates: Defenses Against Viral Entry

Recent genome-wide screening has revealed that the host employs its own structural components as a crucial line of physical defense against viral invasion [13]. This structural immunity appears to manifest as a two-layered physical barrier within the intestinal cells targeted by Orsay virus.

The first barrier is composed of intestinal collagens (e.g., COL-51, COL-61), which are expressed by intestinal cells and are thought to form a protective glycocalyx on the cell surface. This collagen-rich layer appears to function by directly blocking or impeding viral entry into the cell [13]. The second, deeper barrier is the intestinal terminal web, a dense network of cytoskeletal filaments underlying the apical membrane. This network, whose primary component is the intestine-specific actin ACT-5, acts as a sub-apical barricade, further restricting viral ingress and movement [13].

The integrity of this actin-based barrier is dynamically maintained by a suite of actin-remodeling proteins (such as WSP-1 and UNC-34) and is even regulated by epigenetic factors (like NURF-1 and ISW-1), highlighting a coordination between the cell’s structural integrity and its antiviral defense posture [13].

#### 2.1.2. The Intracellular Battlefield: Suppressing Viral Replication and Gene Expression

RNA Interference (RNAi): The Precision-Guided System

**Figure 1:**
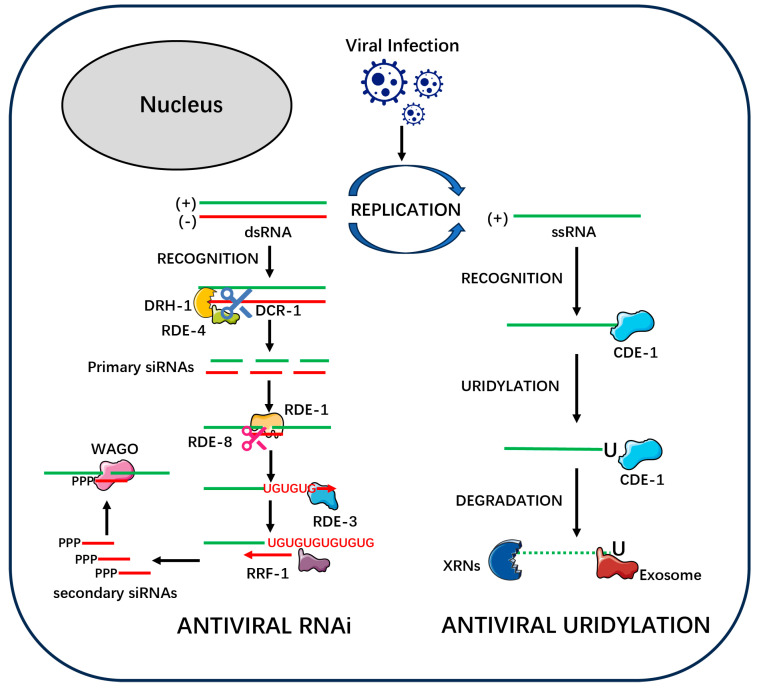
A Molecular Mechanism of Antiviral RNAi and Uridylation Pathways in Nematodes. A schematic overview of the two primary antiviral defense pathways operating in the nematode cytoplasm. Upon viral infection, the host cell mounts a two-pronged defense involving RNA interference (RNAi) and direct uridylation-mediated degradation. On the left, the Antiviral RNAi pathway is depicted as a multi-step process involving amplification. (1) Viral replication produces viral double-stranded RNA (dsRNA), which is recognized by a complex containing the helicase DRH-1, the dsRNA-binding protein RDE-4, and the Dicer nuclease DCR-1. (2) DCR-1 cleaves the viral dsRNA into primary small interfering RNAs (siRNAs), which are approximately 23 nucleotides (nt) in length. (3) A primary siRNA is loaded into the Argonaute protein RDE-1. The RDE-1/siRNA complex then targets complementary viral RNA. (4) RDE-1 recruits the nuclease RDE-8 to cleave the target RNA. (5) This cleavage event triggers a powerful amplification loop. The terminal uridylyltransferase RDE-3 adds a poly(UG) tail to the 3′ end of the RDE-8 cleavage product. (6) The poly(UG) tail serves as a template for the RNA-dependent RNA polymerase RRF-1 to synthesize abundant secondary dsRNAs. (7) These secondary dsRNAs are processed into 22G-RNAs (secondary siRNAs), which characteristically possess a 5′-triphosphate (PPP) group. (8) The 22G-RNAs are loaded into a distinct class of worm-specific Argonaute proteins (WAGOs) to direct widespread silencing of viral transcripts, thus amplifying the antiviral response. On the right, the Antiviral Uridylation pathway functions as a direct degradation mechanism. (1) The terminal uridylyltransferase CDE-1 directly recognizes viral single-stranded RNA (ssRNA). (2) CDE-1 adds a short oligo(U)-tail to the 3′ end of the viral RNA, “tagging” it for destruction. (3) This U-tail is recognized by the cell’s general RNA decay machinery, including 5′-3′ exonucleases (XRNs) and the 3′-5′ Exosome complex, leading to the rapid degradation of the viral genome.

RNA interference (RNAi) is a cornerstone of the nematode antiviral defense network. The discovery of Orsay virus in an RNAi-deficient *Caenorhabditis elegans* mutant provided a powerful model for studying this natural antiviral mechanism [1]. Unlike plants and insects, which utilize multiple Dicer proteins, nematodes possess only a single Dicer. This “single Dicer dilemma” likely drove the evolution of unique strategies to ensure robust antiviral defense without compromising essential miRNA-mediated regulation [17]. This section will deconstruct the nematode antiviral RNAi response into its two core stages, using the well-studied *C. elegans* system as a benchmark while drawing critical comparisons to parasitic nematodes (Figure 1).

Primary siRNA Biogenesis: A Conserved Core with Architectural Variations

In *C. elegans*, antiviral RNAi is initiated by a core complex comprising the Dicer enzyme (DCR-1), the dsRNA-binding protein (RDE-4), and the key helicase DRH-1 [18]. DRH-1 functions as a pattern recognition receptor that surveys the cytoplasm for viral double-stranded RNA (dsRNA), a hallmark of RNA virus replication. Upon detecting these viral RNAs, DRH-1 binds and unwinds the duplex regions, effectively “exposing” them and facilitating their recognition and processing by downstream RNAi components. This action ensures that the viral dsRNAs are made accessible for precise cleavage by the RNase III enzyme DCR-1, which subsequently acts as a “molecular ruler” to generate small interfering RNAs (siRNAs) of defined length [18]. Together, DRH-1 and DCR-1 establish the first line of RNA-based antiviral defense, linking pathogen recognition with effector silencing. A homolog of the mammalian viral sensor RIG-I plays a crucial ‘sentinel’ role by ‘exposing’ viral dsRNAs, making them accessible for cleavage [18]. DCR-1 then acts as a ‘molecular ruler,’ cleaving the viral dsRNA into primary siRNAs of approximately 23 nucleotides (nt) [19].

This Dicer-centric machinery appears to be a fundamental feature of nematode immunity, as core components like DCR-1 and DRH-1 are highly conserved across the phylum [20]. However, comparative genomic surveys reveal critical architectural variations; for instance, the essential Dicer co-factor RDE-4 is notably absent from the genomes of many parasitic species [20]. Despite these architectural gaps, functional evidence confirms the pathway is active in parasitic nematodes. A recent study of *Brugia malayi* infected with its natural virus (BMRV1) demonstrated a strong RNAi response, hallmarked by the production of virus-derived siRNAs (vsiRNAs) that were predominantly 23 nt in length [2].

Once generated in *C. elegans*, primary siRNAs are loaded into the Argonaut protein RDE-1. In a unique division of labor, RDE-1’s intrinsic slicer activity is used only to remove the siRNA passenger strand [21]. For target cleavage, RDE-1 acts as a navigator, recruiting the nematode-specific nuclease RDE-8 to execute the cut [22].

Signal Amplification: A Specialized Innovation Versus a Basal Response

The RDE-8-mediated cleavage in *C. elegans* is not the end of the defense but rather the trigger for a powerful, Dicer-independent amplification loop [17]. This process begins when the terminal transferase RDE-3 adds a special poly(UG) tail to the 3′ ends of the RDE-8 cleavage products [23]. This poly(UG) tail acts as a unique molecular ‘beacon’ that is specifically recognized by the RNA-dependent RNA polymerase (RdRP) RRF-1, which then synthesizes a massive volume of secondary siRNAs [24]. These secondary siRNAs, also known as 22G-RNAs, have distinct biochemical features, including a 5′-triphosphate group and a uniform length of 22 nt, typically starting with a guanine [25,26]. Finally, these newly synthesized secondary siRNAs are loaded onto a large family of worm-specific Argonaute proteins (WAGOs) to silence additional viral RNAs [27].

While RdRP-mediated amplification of RNAi is an ancient defense mechanism found across diverse kingdoms, including in plants and fungi, the specific molecular machinery that governs this process in *C. elegans* appears to be a specialized innovation within the *Caenorhabditis* lineage. Genomic surveys show that key components are poorly conserved or absent in parasites. More strikingly, the nuclear Argonaute NRDE-3, essential for transgenerational inheritance of silencing in *C. elegans*, is completely absent from all surveyed parasitic nematode datasets [20]. This genomic disparity raises the question of whether the secondary amplification machinery is functionally conserved in parasites.

This prediction is compellingly supported by functional data from the B. malayi antiviral response. Analysis of the virus-derived small interfering RNAs (vsiRNAs) targeting BMRV1 revealed limited evidence for the production of abundant secondary siRNAs, such as the 22G-RNAs that characterize the amplified response in *C. elegans* [2]. Taken together, these data suggest that, while many nematodes possess a robust primary RNAi response, they may lack the explosive secondary amplification machinery.

CDE-1: The Uridylation “Tagging” Weapon

In the nematode antiviral ‘armory,’ if RNA interference (RNAi) is the ‘precision-guided system,’ then the uridylation pathway mediated by the terminal uridylyltransferase CDE-1 represents an equally critical, yet mechanistically distinct, direct-acting weapon. This pathway operates independently of RNAi, ‘tagging’ viral RNA to direct its rapid clearance by cellular degradation machinery, thus forming a parallel line of defense [14] (Figure 1).

The antiviral function of CDE-1 was first identified through a forward genetic screen for host factors conferring resistance to Orsay virus (OrV). CDE-1 is a terminal uridylyltransferase (TUT) and a homolog of the mammalian TUT4 and TUT7 proteins, which are known to regulate mRNA stability [14,28]. Its antiviral role is dependent on its catalytic activity and is executed through a two-step “tag-and-degrade” process. First, CDE-1 recognizes the viral RNA and adds a short U-tail, typically consisting of one or two uridines, to its 3′ end. This U-tail serves as a molecular “degradation tag” that is recognized by the cell’s generic RNA decay machinery, including 5′-3′ exonucleases of the XRN family and the 3′-5′ exosome complex, which then rapidly and completely degrade the tagged viral RNA [14].

It is crucial to emphasize that CDE-1-mediated antiviral uridylation is a fundamentally different process from the uridylation that occurs within the RNAi amplification loop described in the previous Section 2.1.2. The key distinctions are as follows:

**Different Core Enzymes:** The primary antiviral uridylation is executed by the terminal uridylyltransferase CDE-1 [14]. In contrast, the modification within the RNAi pathway is performed by RDE-3 [17].
**Different RNA Substrates:** CDE-1 acts directly on the viral single-stranded RNA (ssRNA) genome. Conversely, the RDE-3 in the RNAi pathway acts on viral RNA fragments that have been previously cleaved by the nuclease RDE-8 [17].
**Different Chemical Modifications:** CDE-1 adds a simple, short U-tail (1-2 uridines) to its target. In stark contrast, RDE-3 adds a structurally distinct poly(UG) tail, composed of alternating uridine and guanosine nucleotides [23].
**Different Functional Purposes:** The purpose of the CDE-1-mediated U-tail is to directly mark the viral RNA for degradation, serving as a terminal signal that operates independently of the RNAi pathway [28]. The purpose of the RDE-3-generated poly(UG) tail, however, is to create a unique molecular beacon that serves as a template for the RNA-dependent RNA polymerase (RdRP) RRF-1 to amplify the production of secondary siRNAs within the RNAi pathway [24].

Importantly, this CDE-1-mediated pathway appears to be a deeply conserved branch of nematode immunity. A comparative genomics study identified clear orthologs of cid-1 (the gene encoding CDE-1) across diverse nematode clades, including key animal parasites like *Ascaris suum* and *Brugia malayi*, as well as the significant plant parasite *brugia dogyne incognita* [20]. The broad conservation of this key enzyme strongly suggests that this direct “tag-and-degrade” mechanism is a fundamental layer of antiviral defense across the phylum.

Moreover, this CDE-1-mediated defense strategy is not unique to nematodes but is an evolutionarily conserved, ancient mechanism. The mammalian homologs of CDE-1, TUT4 and TUT7 have also been shown to uridylate the mRNA of Influenza A Virus (IAV), thereby inhibiting viral protein expression and limiting viral replication in the early stages of infection [14].

The Intracellular Pathogen Response (IPR): A Fortress Defense

**Figure 2:**
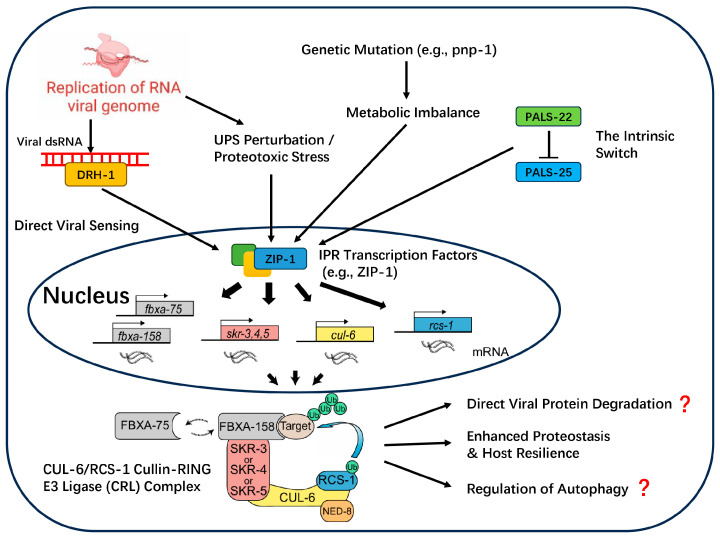
This model depicts four parallel pathways that activate the Intracellular Pathogen Response (IPR) in *C. elegans*. First, upon viral infection, direct sensing of viral replication intermediates, such as double-stranded RNA (dsRNA), is mediated by the RIG-I-like receptor DRH-1. This DRH-1-mediated activation is specific to viral infection and functions independently of the canonical RNAi machinery to induce IPR gene transcription. Second, viral replication can also cause proteotoxic stress by overwhelming the Ubiquitin–Proteasome System (UPS), which serves as a distinct trigger for the IPR. Third, metabolic imbalance, such as that caused by mutations in the purine salvage pathway gene pnp-1, can independently activate the IPR. Fourth, an intrinsic switch composed of the antagonistic paralogs PALS-22 (a repressor) and PALS-25 (an activator) regulates IPR activation independently of external threats. These parallel signals are proposed to converge in the nucleus to activate IPR-specific transcription factors, such as the central activator ZIP-1. This leads to the upregulation of a suite of IPR effector genes, including components of a Cullin-RING E3 Ligase (CRL). In the cytoplasm, these components assemble into the CUL-6/RCS-1 CRL complex. This effector complex is thought to execute antiviral functions through several mechanisms, including enhancing host proteostasis and resilience, and potentially through the direct degradation of viral proteins or regulation of autophagy (indicated by question marks).

The Intracellular Pathogen Response (IPR) is a hallmark defense program that, based on current knowledge, appears to be a specialized innovation that has evolved in *C. elegans* to specifically combat intracellular threats. Our current molecular understanding of the IPR has been primarily elucidated using the *C. elegans*–Orsay virus pathosystem, which serves as the canonical model for studying this pathway [1]. As illustrated in Figure 2, the IPR constitutes a complete signaling cascade, beginning with the perception of diverse danger signals, processing through downstream transcription factors, and culminating in the activation of an effector module centered on ubiquitination to enhance host proteostasis [29].

Activation of the IPR: A Multi-Trigger, Parallel Input System

The IPR is not activated by a single linear pathway, but rather by at least four independent, parallel pathways that sense different danger signals. This multi-trigger architecture provides robust and layered protection.

The most direct activation route involves the recognition of Pathogen-Associated Molecular Patterns (PAMPs) by the RIG-I homolog helicase DRH-1. A key study demonstrated that the expression of a replication-competent Orsay virus RNA1 segment, which encodes the viral RNA-dependent RNA polymerase (RdRP), was sufficient to activate the entire IPR; this activation was lost if the polymerase was rendered catalytically inactive [30]. This provides strong evidence that DRH-1 specifically recognizes molecular patterns generated during viral replication to initiate the downstream IPR transcriptional program, a function independent of its role in RNAi [30].

The IPR system can also infer threats by sensing disruptions in core host metabolic pathways, particularly the purine salvage pathway, where enzymes like ADAH-1 and PNP-1 act as checkpoints [31,32]. The abnormal accumulation of the endogenous metabolite deoxyadenosine serves as a Danger-Associated Molecular Pattern (DAMP) that robustly activates the IPR, independently of the DRH-1 viral sensing pathway [31].

Finally, the IPR responds to proteotoxic stress resulting from viral replication overwhelming the host’s Ubiquitin–Proteasome System (UPS). Direct pharmacological or genetic inhibition of the UPS is sufficient to induce IPR gene expression, establishing “UPS stress sensing” as a third independent activation pathway that does not require DRH-1 [30,33].

Distinct from pathways that sense external or indirect threats, the IPR can also be triggered by an intrinsic “state-gating” switch composed of two antagonistic paralogs, PALS-22 and PALS-25. Under normal conditions, the repressor PALS-22 is active, keeping the IPR off. However, the loss of PALS-22 function is sufficient on its own to activate PALS-25, turning on the IPR and shifting the animal into a “pro-defense” state. This intrinsic module represents a key internal decision point for orchestrating immunity, and its central role in managing the fundamental trade-off between defense and host fitness will be deconstructed in the regulatory layer (Section 2.2.2).

The Effector Machinery and Speculative Antiviral Mechanisms

As depicted in Figure 1 and Figure 2, regardless of the activation route, these various signals appear to converge on downstream transcription factors that ultimately upregulate a common effector module. The best-characterized of these is a specific Cullin-RING E3 ubiquitin ligase (CRL) complex. This complex consists of the CUL-6 scaffold, the RING protein RCS-1, redundant Skp-related adaptors (SKR-3/4/5), and substrate-recognition receptors (FBXA-75 and FBXA-158) [15,34].

This CRL complex enhances host proteostasis, conferring thermotolerance and reducing protein aggregation [15]. Despite the lack of direct experimental verification, the potential antiviral mechanisms are hypothesized to include:

**Direct degradation of viral proteins:** A logical function for an E3 ligase in an antiviral response is to directly target viral components for degradation [15,34].

**Regulation of other immune pathways:** Drawing analogy from the function of the human homolog of RCS-1, TRIM23, the RCS-1/CUL-6 complex might regulate other cellular processes like autophagy to inhibit viruses [35].

**Enhancement of host resilience:** By clearing damaged host proteins, the IPR effector module helps the cell tolerate the stress of infection, a function directly supported by proteostasis assays in *C. elegans* [15].

Cross-functional Immunity: Repurposing Core Cellular Machinery

Beyond dedicated immune pathways, the nematode defense network also displays remarkable wisdom by repurposing key proteins from other fundamental cellular programs. A prominent example comes from the Programmed Cell Death (PCD) pathway. A pioneering study using an artificial infection model with Vaccinia Virus found that viral replication in the intestine and other tissues was significantly enhanced in *C. elegans* mutants lacking the core PCD executioners, CED-3 or CED-4 [16]. Crucially, this antiviral activity was demonstrated to be an independent, non-canonical molecular function, separate from apoptosis itself [16]. Although the study used an artificial infection model with Vaccinia Virus (VV), a mammalian virus not known to naturally infect nematodes, it provided the critical proof-of-concept that these core cell-death proteins can “moonlight” as direct viral restriction factors [16]. Given that orthologs of these apoptotic proteins are highly conserved across the animal kingdom, including in parasitic nematodes, this raises the intriguing possibility that this moonlighting function may represent a deeply conserved layer of innate immunity, although its role during natural viral infections remains to be explored [16].

This principle of “weapon borrowing” also blurs the traditional lines between antibacterial and antiviral immunity. Recent transcriptomic studies have revealed that classic Antimicrobial Effectors (AMEs) are recruited to the antiviral battlefield [11]. For example, the lysozyme lys-2 is rapidly upregulated upon Orsay virus infection and functionally restricts viral spread. However, the arms race is complex, as other proteins like the C-type lectin clec-60 appear to be co-opted by the virus to play a pro-viral role [11]. Since the genomes of parasitic nematodes are also rich in these diverse effector families, it is plausible that they represent a “functional reservoir” that can be similarly repurposed or hijacked during viral infections.

### 2.2. The Second Tier: The Command and Control Network of Trade-Offs

It is crucial to note that our current, deep understanding of the regulatory architecture of nematode immunity is almost exclusively derived from studies in the model organism *C. elegans*. Research in this tractable system has revealed that, while the effector arsenal provides the firepower, activating these powerful pathways is metabolically expensive and can cause collateral damage [11], creating a fundamental trade-off between defense and essential life processes like growth and reproduction. The nematode’s regulatory network is the solution to this challenge, acting as a hub that integrates internal physiological state with external threat signals to orchestrate a proportional and cost-effective defense.

Intriguingly, the principles of this cost–benefit management are not unique to nematodes. The regulatory logic of the nematode’s Intracellular Pathogen Response (IPR) is conceptually analogous to the vertebrate type I interferon (IFN-I) response [36]. This parallel suggests that nematodes and vertebrates may have convergently evolved similar strategies to manage the universal challenge of immune homeostasis. Therefore, this section will deconstruct the architecture of this regulatory network, primarily using the *C. elegans* model, to illustrate how the host–virus truce is maintained.

#### 2.2.1. The Rationale for Regulation: The Inevitable Cost of an Antiviral Response

The principle that “immunity is costly” is substantiated by multi-level evidence in the nematode antiviral system, highlighting the absolute necessity for tight regulation.

**Metabolic and Physiological Costs:** The clearest evidence for this cost comes from the constitutive activation of the immune system, which severely impairs host fitness even in the absence of a pathogen. For example, in mutants of the key IPR repressor gene *pals-22*, the worm gains powerful resistance to viruses but pays a steep price in the form of slowed development and a significantly shortened lifespan [29]. Likewise, in mutants of another repressive transcription factor, *sta-1*, the constitutively active antiviral genes also lead to a shortened lifespan [37]. However, this fitness cost cannot be attributed solely to immune hyperactivation due to the other physiological processes regulated by these key repressor genes. This cost–benefit picture becomes more complex during an active infection, where it is challenging to deconvolve the cost of immunity from direct viral pathology. For instance, dynamic transcriptomic studies of an ongoing viral infection paint a clear picture of massive physiological disruption, triggering a large-scale reprogramming of host metabolism, such as the strategic downregulation of genes related to lipid metabolism and fatty acid elongation during peak viral replication [11]. Besides, it was observed that lipid abundance was significantly decreased in infected worms, which supported the cost of the immune activation. However, this specific metabolic shift could represent a strategic host resource reallocation (a cost), but it is also plausible that it is a pathology induced by the virus to promote its own replication.
**Evolutionary Cost:** This fundamental trade-off is so profound that it has left an indelible mark on the species’ genome. Population genetic analyses of wild *C. elegans* isolates revealed that the genomic locus containing the core IPR regulators, pals-22 and pals-25, is under strong balancing selection [38]. Direct evidence for this includes the atypically high Tajima’s D values for multiple pals genes, which indicates that evolution has actively maintained multiple different versions (haplotypes) of this immune switch in the population, rather than selecting a single “best” version [38]. This finding provides powerful evidence that the cost–benefit analysis of immunity is a dynamic challenge optimized at the evolutionary scale, with different strategies (e.g., “always ready” versus “induce on-demand”) being favored in different pathogenic environments [38].

#### 2.2.2. The Command Hierarchy: A Multi-Input Regulatory Decision System

To manage the immense cost of immunity and mount a precise and proportional defense, the nematode has evolved a command hierarchy. This system operates through two principal strategies: Direct Threat-Responsive Mechanisms that are triggered by the pathogen itself, and Global State-Dependent Modulation, where the host’s overarching physiological condition pre-determines its capacity to respond.

**Direct Threat-Responsive Mechanisms**

**Figure 3:**
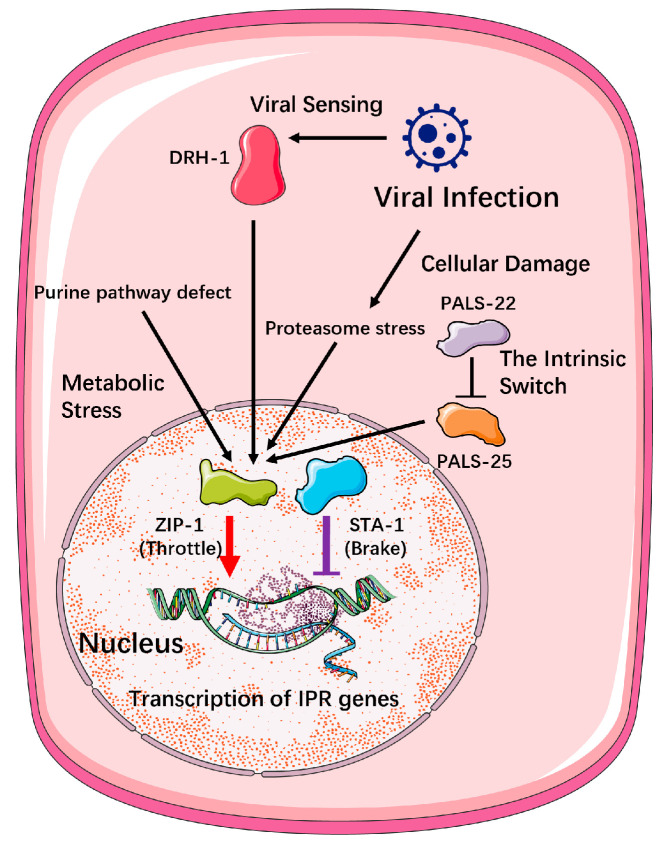
Transcriptional Regulation of the Nematode Intracellular Pathogen Response (IPR). The nematode IPR is controlled by a “throttle-and-brake” system in the nucleus. Four distinct upstream pathways, including direct viral sensing (DRH-1), metabolic stress, cellular damage, and an intrinsic switch (PALS-22/25), respond to viral infection [36,37,38,39]. These signals converge to activate the transcription factor ZIP-1 (the “throttle”), which promotes IPR gene expression. This activation is opposed by the repressor protein STA-1 (the “brake”), which limits the response. The balance between ZIP-1 and STA-1 activity fine-tunes the host’s antiviral defense. Standard arrows (→) indicate activation or direct processes. Blunt-ended lines (—|) indicate direct inhibition.

When a virus invades, a suite of immediate and highly specific regulatory mechanisms is deployed to fine-tune the defensive output. A prime example of the nematode’s regulatory logic is the intrinsic ‘state-gating’ switch composed of the antagonistic paralogs PALS-22 and PALS-25 (Figure 3). However, this regulatory module appears to be a relatively recent evolutionary innovation, deeply rooted in the species-specific expansion of the pals gene family within the *Caenorhabditis* genus [29].

While *C. elegans* possesses 39 pals genes, its close relative C. briggsae has only 8 [29]. More importantly, detailed comparative analyses have shown that no clear orthologs of the key switch components, pals-22 and pals-25, exist in C. briggsae or 26 other surveyed nematode species [29]. This module serves as the central arbiter of a fundamental trade-off in *C. elegans*, toggling the animal’s physiology between a default “pro-growth” program and a costly “pro-defense” program. Under normal conditions, the repressor PALS-22 is active, inhibiting the activator PALS-25 to maintain homeostasis and prioritize development.

Crucially, this module represents a distinct and independent route to activating the Intracellular Pathogen Response (IPR) in *C. elegans*. The loss of PALS-22 function is, on its own, sufficient to engage a full-scale defense response through PALS-25, establishing it as a key internal checkpoint. This intrinsic activation pathway operates in parallel to the threat-sensing mechanisms discussed previously; it does not require input from the viral sensor DRH-1, nor from the pathways that detect metabolic or proteotoxic stress [29].

Therefore, the PALS-22/25 switch exemplifies how the defense network is controlled not only by external threat detection but also by internal, pre-programmed regulatory circuits that weigh the profound fitness costs of immunity against the benefits of growth. Its absence in most other nematodes strongly suggests that these organisms must have evolved alternative “command and control” networks to manage this crucial trade-off [29].

**Global State-Dependent Modulation**

**Figure 4:**
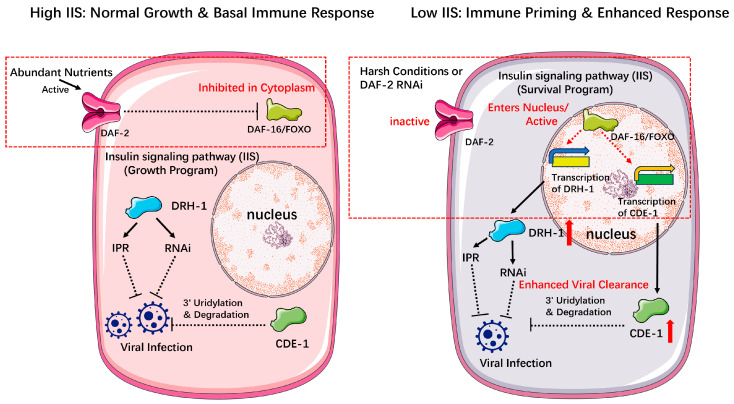
A Model for the Regulation of Antiviral Immune Readiness by the Insulin/IGF-1 Signaling (IIS) Pathway. This model illustrates how the IIS pathway acts as a global modulator, shifting *C. elegans* between a ‘growth’ state with basal immunity and a ‘survival’ state with a primed and enhanced antiviral response. The model is based on findings demonstrating that reduced IIS conditionally upregulates antiviral effectors upon infection [41]. (**Left**) High IIS: Normal Growth & Basal Immune Response. Under favorable conditions, such as abundant nutrients, the DAF-2 receptor is active. This leads to the inhibition of the transcription factor DAF-16/FOXO, which is sequestered in the cytoplasm, promoting a “Growth Program”. Upon viral infection, antiviral effectors such as DRH-1 (which activates RNAi and IPR) and CDE-1 (which mediates 3′ uridylation and degradation) mount a basal immune response, leading to normal viral clearance. (**Right**) Low IIS: Immune Priming & Enhanced Response. Under harsh conditions or experimental *daf-2* RNAi, the DAF-2 receptor is inactive. This allows DAF-16/FOXO to translocate to the nucleus and become active, shifting the animal into a “Survival Program” that includes immune priming. While this state does not constitutively activate immune genes, upon viral infection, active nuclear DAF-16 is proposed to drive the potent upregulation of drh-1 and cde-1 transcription. This results in higher levels of DRH-1 and CDE-1 protein, leading to an enhanced antiviral response and more efficient viral clearance, without an associated fitness cost. Standard arrows (→) indicate activation or direct processes. Dotted blunt-ended lines (····|) indicate indirect or multi-step inhibition. Red dashed arrows (--►) represent an inferred regulatory link that is strongly supported by correlational data but where direct causality has not been formally demonstrated.

Distinct from the direct responses to viral invasion, the nematode’s fundamental capacity to fight infection is profoundly modulated by its overarching physiological state. The Insulin/IGF-1 Signaling (IIS) pathway is an evolutionarily conserved hub that functions as an environmental sensor and a life-strategy rheostat in *C. elegans*. When environmental conditions are favorable and food is abundant, the IIS pathway is active, promoting normal growth and reproduction. However, in response to survival threats such as food scarcity or high temperature, IIS pathway activity is down-regulated, shifting the worm’s physiology from a “growth program” to a “survival program” centered on longevity and stress resistance, such as entry into the dauer diapause [40]. When the pathway is active, the key effector transcription factor DAF-16/FOXO is inhibited and sequestered in the cytoplasm (Figure 4). Conversely, reduced IIS activity—as occurs in harsh environments—allows DAF-16 to translocate to the nucleus and drive its target gene expression programs (Figure 4) [40].

This classic survival-regulating pathway has recently been shown to be deeply intertwined with antiviral defense. Experimentally reducing IIS in adulthood, for example, through *daf-2* RNAi, primes the immune system into a state of heightened readiness [41]. The elegance of this mechanism lies in its “threat-dependent” activation. In the absence of a virus, this reduced IIS-induced (rIIS)-induced “primed” state does not trigger a costly constitutive upregulation of antiviral genes. However, upon viral infection, the primed state enables a potent transcriptional counter-attack [41]. This enhanced, conditional immune response leads directly to lower viral loads and increased survival under infection, without an associated reproductive trade-off [41]. This demonstrates that the IIS pathway functions as a master strategist, setting the rules of engagement based on long-term physiological context to maximize survival at minimal cost (Table 1).

The power of this regulatory principle lies in its deep evolutionary conservation. The core components of the Insulin/IGF-1 Signaling pathway, including orthologs of the DAF-2 receptor and the DAF-16/FOXO transcription factor, are highly conserved in diverse parasitic nematodes [42,43,44,45]. Crucially, this conservation extends to function. Orthologs of DAF-16 from parasites like *Haemonchus contortus* and *Strongyloides stercoralis* are not only structurally similar but have been shown to be functionally active, regulating the development of infective larvae in a manner analogous to dauer formation in *C. elegans* [42,43]. In fact, the *H. contortus* DAF-16 can even restore dauer formation to *daf-16* mutant worms [43]. Likewise, the DAF-2 receptor from *H. contortus* shows conserved structure and can partially rescue *daf-2* mutants [44]. This deep conservation of the IIS pathway’s role in orchestrating life-history decisions raises the compelling hypothesis that parasitic nematodes also leverage this system to link their metabolic state with immune readiness, representing a universal logic for managing immune costs across the phylum.

## 3. Summary and Outlook

### 3.1. Summary: The Architecture of a Negotiated Truce

This review has deconstructed a central paradox of modern nematode virology: the stark contrast between a pervasively virus-rich world and the rarity of severe, overt disease. We have argued that this delicate “host–virus truce” is not a passive stalemate but an actively managed state, arbitrated by a multi-layered host immune network. The first tier of this network, the “effector layer,” comprises a diverse arsenal of direct-acting weapons. This includes the precision-guided RNA interference (RNAi) system, the parallel CDE-1-mediated ‘tag-and-degrade’ pathway, the broad transcriptional shield of the Intracellular Pathogen Response (IPR), and even repurposed components from core cellular programs like programmed cell death and the cytoskeleton, forming a multi-pronged defensive front.

However, we posited that the true wisdom of nematode immunity lies not in this raw firepower but in the second tier: the “regulatory layer.” The activation of these powerful effectors incurs significant metabolic and physiological costs, creating a fundamental trade-off between defense and host fitness. This command-and-control network acts as a decision-making hub. Through intrinsic switches like the PALS-22/25 module, and global state-sensors like the Insulin/IGF-1 Signaling pathway, it integrates diverse internal and external cues to orchestrate a proportional, cost-effective defense. It is the dynamic interplay between this potent effector arsenal and its judicious regulatory command that allows nematodes to navigate the constant threat of infection. This manifests as different survival strategies—from the “controlled pathogenicity” in *C. elegans* to the enigmatic “persistent tolerance” in many parasites—ultimately preserving the host’s evolutionary fitness in the face of pervasive viral challenge.

### 3.2. Outlook: Unraveling the Secrets of the Truce

The conceptual framework of a two-tiered immune network, balanced by cost–benefit analysis, provides a powerful new lens through which to interpret the diverse outcomes of nematode-virus interactions. However, this framework, built largely upon the powerful genetics of the *C. elegans* model system, also illuminates vast and exciting frontiers of discovery. The central challenge for the field is now to move beyond this foundational model, to dissect the molecular strategies employed across the vast diversity of the nematode phylum, and to understand the broader ecological and medical implications of this ancient host–virus arms race. Looking ahead, several key questions and research avenues emerge as particularly critical for unraveling the remaining secrets of this delicate truce:

#### 3.2.1. The Arms Race Full Picture: Viral Counter-Weapons and Host Back-Up Defenses

The co-evolution of viruses and hosts is a perpetual arms race. A major gap in our current understanding is the nature of the viral “counter-weapons” used to disarm nematode immunity. A prime suspect for viral attack is the host’s ubiquitin–proteasome system. In human virology, it is a classic strategy for viruses to hijack host Cullin-RING E3 ligases (CRLs) to eliminate antiviral factors. The HIV-1 Vif protein, for instance, hijacks the CUL5-E3 ligase complex to degrade the host restriction factor APOBEC3G [46], a process recently shown to be even more complex, involving the recruitment of a second E3 ligase, ARIH2, to form a highly efficient degradation “tag-team” [47]. Given that the core of the nematode IPR pathway is the CUL-6/CUL5-based E3 ligase, it is highly plausible that nematode viruses have evolved analogous strategies to hijack this central hub. Future studies using proteomics to screen for viral proteins that interact with the CUL-6 complex will be crucial to test this hypothesis.

Conversely, as viruses evolve to suppress primary defenses like RNAi, hosts must evolve backup systems. The existence of RNAi-independent antiviral defenses (RIADs) in nematodes has long been hypothesized, but direct evidence was scarce. A recent targeted genetic screen by Xia et al. [48] provided the first systematic proof, identifying several new RIAD genes. Crucially, they demonstrated that, in an RNAi-deficient background, the loss of one of these genes, riad-1, turned a mild Orsay virus infection into a lethal one. This highlights that RIAD is not a minor pathway but a vital “last line of defense.” A key future direction will be to elucidate the molecular functions of these new RIAD genes and to explore their conservation and roles in parasitic nematodes.

#### 3.2.2. Beyond *C. elegans*: Deciphering the Immune Logic and Application Potential in Parasitic Nematodes

Extending our deep mechanistic understanding from *C. elegans* to the vast and medically important world of parasitic nematodes is a paramount challenge. This review has highlighted potentially crucial differences in their immune toolkits. For instance, genomic and functional data suggest that the explosive secondary RNAi amplification machinery, so central to *C. elegans* defense, may be diminished or absent in many parasites. This difference could be a key factor contributing to the “persistent tolerance” phenotype, where an immune response is sufficient to control a virus and prevent disease but not to eradicate it, leading to a stable, long-term truce. Similarly, while core effector pathways are conserved, the regulatory architecture appears divergent. How do parasitic nematodes, lacking key regulators like the PALS-22/25 switch, manage the critical trade-off between defense and fitness? The conserved Insulin/IGF-1 Signaling (IIS) pathway, a master regulator of life-history decisions, is a strong candidate for this role and warrants deep functional investigation.

Furthermore, understanding these immune mechanisms has profound practical implications. The successful genetic manipulation of parasitic nematodes, crucial for functional genomics and drug target validation, is often hampered by transgene silencing, a process driven by the host’s RNAi machinery. The groundbreaking work of Hagen et al. [49], which utilized a lentiviral vector to successfully deliver RNAi-inducing cassettes into the animal parasite *Nippostrongylus brasiliensis*, demonstrates that viral vectors can overcome these barriers [49]. This opens a path to designing “immune-stealth” vectors for more efficient gene editing. Conversely, for biological control, this knowledge can be inverted. By engineering viruses to carry potent inhibitors of nematode immune pathways (e.g., RNAi or RIAD), we could develop “smarter” and more effective viral biocontrol agents to combat agricultural pests.

#### 3.2.3. The “Trojan Horse” on a Bigger Stage: The Impact of the Nematode Virome on Final Hosts

The internal immune struggle within the nematode has external consequences that extend to the broader medical landscape. The concept of a parasite acting as a “Trojan Horse” to introduce its own microbiome into a final host is gaining significant traction. While co-infection studies have shown that a helminth infection can systemically alter a host’s susceptibility to other unrelated viruses, the direct impact of viruses carried within the parasite is a more intimate and potentially more potent interaction [50].

The work of Williams et al. [12] elegantly demonstrated this physical reality, using in situ hybridization to show that novel viruses discovered in mouse liver tissue were, in fact, located exclusively within parasitic nematodes residing there. Building on this, the landmark study by Quek et al. [2] provided the first immunological proof of this interaction’s relevance. They found that humans infected with the filarial nematode *Onchocerca volvulus* develop specific antibodies not just against the worm, but also against the OVRV1 virus it carries. This unequivocally demonstrates that the nematode virome is “visible” to the human immune system. This raises a critical and unexplored question: Do these viral components, released during infection, act as novel PAMPs that modulate the host’s immune response to the parasite itself, thereby influencing the clinical outcome of the parasitic disease? Answering this question will be a crucial next step in understanding the full complexity of host–parasite interactions and may open new avenues for diagnosis and therapeutic intervention.

## Figures and Tables

**Table 1:** Summary of the Two-Tiered Architecture of Nematode Antiviral Defense.

Tier	Component/Pathway	Key Proteins (Examples)	Primary Function & Role
Tier 1: The Effector Layer	Structural Barriers	COL-51, COL-61, ACT-5	Physical “barricades” (e.g., collagens, actin) that block or impede viral entry and ingress into intestinal cells.
	RNA interference (RNAi)	DCR-1, DRH-1, RDE-1, RRF-1, WAGOs	A “precision-guided system” that cleaves viral dsRNA (primary siRNAs) and uses RdRPs for a powerful amplification loop (secondary siRNAs).
	Uridylation Pathway	CDE-1	An RNAi-independent “tag-and-degrade” mechanism; CDE-1 adds a U-tail to viral ssRNA to mark it for destruction by host exonucleases.
	Intracellular Pathogen Response (IPR)	ZIP-1, CUL-6/RCS-1 (CRL Complex)	A broad “fortress defense”; a transcriptional program that upregulates an effector module (E3 ligase) to enhance host proteostasis and resilience.
	Repurposed Core Machinery	CED-3, CED-4 (PCD); lys-2 (AMEs)	“Moonlighting” proteins from other core systems (e.g., Programmed Cell Death, antibacterial effectors) are recruited for antiviral functions.
Tier 2: The Regulatory Layer	Intrinsic “State-Gating” Switch	PALS-22 (Repressor), PALS-25 (Activator)	An internal “pro-growth” vs. “pro-defense” toggle that manages the high fitness costs of constitutive immunity by keeping the IPR off in the absence of threat.
	Global State-Dependent Modulation	Insulin/IGF-1 Signaling (IIS) Pathway (DAF-2, DAF-16)	A “life-strategy rheostat” that links the host’s metabolic/environmental state (“growth” vs. “survival”) to immune readiness, “priming” the host for a potent, cost-effective response upon infection.

This table summarizes the key components, proteins, and functions of the “Effector Layer” (Tier 1) and “Regulatory Layer” (Tier 2) of the nematode immune network. This framework is deconstructed and discussed in detail in Section 2.1 (The Effector Layer) and Section 2.2 (The Regulatory Layer).

## Abbreviations

The following abbreviations are used in this manuscript:

AME	Antimicrobial Effector
AMEs	Antimicrobial Effectors
CRL	Cullin-RING E3 Ligase
CRLs	Cullin-RING E3 Ligases
DAMP	Danger-Associated Molecular Pattern
dsRNA	double-stranded RNA
IAV	Influenza A Virus
IFN-I	type I interferon
IIS	Insulin/IGF-1 Signaling
IPR	Intracellular Pathogen Response
OrV	Orsay Virus
PAMPs	Pathogen-Associated Molecular Patterns
PCD	Programmed Cell Death
RdRP	RNA-dependent RNA polymerase
RIAD	RNAi-independent antiviral defense
RNAi	RNA interference
TUT	Terminal Uridylyltransferase
UPS	Ubiquitin–Proteasome System
VV	Vaccinia Virus
vsiRNA	virus-derived siRNA
WAGO	worm-specific Argonaute protein
